# ﻿First record of *Tamarixiadahlsteni* Zuparko (Hymenoptera, Eulophidae), a parasitoid of *Triozaeugeniae* Froggatt (Hemiptera, Triozidae) and current status of the *Tamarixia* species in Mexico

**DOI:** 10.3897/zookeys.1129.90577

**Published:** 2022-11-11

**Authors:** Kenzy I. Peña-Carrillo, Antonio Rodríguez-Rivas, Sara G. Díaz-Ramos, Mayra A. Gómez-Govea, Patricia Zambrano-Robledo, Iram P. Rodríguez-Sánchez, María de Lourdes Ramírez-Ahuja

**Affiliations:** 1 INIFAP, Campo Experimental General Terán, km 31 carretera Montemorelos-China, 67400, General Terán, N.L. Mexico; 2 Universidad de Guadalajara, Centro Universitario de Ciencias Exacta e Ingenierías, Departamento de Madera Celulosa y Papel, Km 15.5, Autopista Guadalajara-Nogales, 45020 Jalisco, Mexico; 3 Universidad Autónoma de Nuevo León, Facultad de Ciencias Biológicas, Laboratorio de Fisiología Molecular y Estructural. Av. Universidad s/n Cd. Universitaria, San Nicolás de los Garza, NL 66455, Mexico; 4 Universidad Autónoma de Nuevo León, FIME-Centro de Investigación e Innovación en ingeniería Aeronáutica (CIIIA), Av. Universidad s/n, Ciudad Universitaria, San Nicolás de los Garza 66455, Mexico

**Keywords:** Biological control, COI, eugenia psyllid, parasitic wasps

## Abstract

Parasitic wasps of the genus *Tamarixia* represent important biological control agents of members of the true bug group, Psylloidea, and are host specific; therefore, they can be used to control insect pests. In this study we report, for the first time, the presence of the parasitoid *Tamarixiadahlsteni* in Mexico and its mitochondrial barcode region of the cytochrome oxidase I gene (COI). We also review the species diversity of the genus *Tamarixia* in Mexico.

## ﻿Introduction

Biological control agents represent a sustainable pest management option that help to maintain pest populations under accepted levels (Wang et al. 2019; Al-Ani et al. 2020). Parasitic wasps from the family Eulophidae represent a very important group of biological control agents as they have a wide range of insect hosts with different degrees of specialization. The genus *Tamarixia* Mercet represents one example of parasitoids with a high degree of host specificity (Urbaneja-Bernat et al. 2019). Most species act as ectoparasitoids, but in some cases endoparasitism has been reported (Noyes 2022). Species of the genus *Tamarixia* parasitize free-living and gall-forming species of true bugs, Psylloidea. It was proposed that they reached this host restriction through a specialization on their host (LaSalle 1994), although the species has also been reported parasitizing aphids (Zuparko et al. 2011). According to the Universal Chalcidoidea database, the genus *Tamarixia* comprises 54 species to date, which are distributed worldwide (Noyes 2022).

In Mexico, five species (both native and exotic) of *Tamarixia* are found: *Tamarixiaaguacatensis* Yefremova (Yefremova et al. 2014), *Tamarixialeucaenae* Boucek (McClay 1990), *Tamarixiaradiata* (Waterston) (González-Hernández et al. 2009), *Tamarixiaschina* Zuparko (Zuparko et al. 2011), and *Tamarixiatriozae* (Burks) (Lomelí-Flores and Bueno 2002). Some of these species have already been part of integrated pest management strategies with remarkable results. For instance, *T.triozae*, the parasitoid of the potato psyllid*Bactericeracockerelli*, was introduced to New Zealand for the biological control of the psyllids that vector the bacterium *Candidatus* Liberibacter *solanacearum* (CLso) (Workman and Whiteman 2009). This bacterium has been linked to different diseases in plants of the nightshade family Solanaceae (Munyaneza et al. 2007). In Mexico, *T.triozae* was found naturally in tomato crops and, according to parasitism evaluation, the percentage of parasitism reached by *T.triozae* on *B.cockerelli* has been up to eighty percent when insecticides are not used to control the psyllid populations (Lomelí-Flores and Bueno 2002). *Tamarixiatriozae* is commercially available in Mexico through Koppert Mexico, and several studies have been carried out on its biological cycle (Rojas et al. 2015), its release into the environment either individually or in combination with other natural enemies for the control of *B.cockerelli* (Cerón-González et al. 2014; Ramírez-Ahuja et al. 2017). Other examples of *Tamarixia* species used for biological control are *T.schina*, which was introduced in California for the control of *Calophyaschini* Tuthill (Psyllidae: Calophyidae), *T.dahlsteni* Zuparko, which was introduced for the control of *Triozaeugeniae* Froggatt (Hemiptera: Triozidae) (Zuparko et al. 2011) and *T.radiata*. The latter is native to Pakistan (Chen and Stansly 2014), but has been introduced into countries such as Taiwan, the United States and France (Guadeloupe), to control populations of the psyllid*Diaphorinacitri* Kuwayama (Hemiptera: Liviidae), a vector of the bacterium *Candidatus* Liberibacter *asiaticus* (Chien et al. 1989; Michaud 2002; De León and Sétamou 2010). In Texas, a reduction of more than ninety percent of *D.citri* populations has been observed in regions where *T.radiata* was released (Flores and Ciomperlik 2017). In Mexico, the parasitoid was reported as an accidentally introduced species (De León and Sétamou 2010).

Currently, DNA barcodes are important tools for species identification with potential for bio-surveillance programs in agriculture (Ashfaq and Hebert 2016). DNA barcodes have been useful to identify important arthropod pests even at immature stages (Ashfaq and Hebert 2016). The common barcoding method used for animal identification is based on the sequencing of a part of the mitochondrial gene cytochrome oxidase subunit I (COI). COI has been used to create universal and public databases of sequences, such as the Barcode of Life Data System (BOLD), which includes agriculturally important insect sequences (Hebert et al. 2003). In this regard, here we report the occurrence of *T.dahlsteni* in Mexico for the first time, and the first mitochondrial cytochrome oxidase subunit one (COI) sequences for this species. We also discuss the potential of additional species of *Tamarixia* in Mexico.

## ﻿Materials and methods

### ﻿Biological samples

We obtained parasitoids emerged from nymphs of *Triozaeugeniae* feeding on *Syzygiumpaniculatum*, collected in an urban area from Zapopan, Jalisco, Mexico [Colonia Las Palomas, Tesistán (20.7890, -103.4831) and Club Deportivo UdeG (20.7793, -103.6075)]. The nymphs were taken to the laboratory (HR 70%, T 25 ± 2 °C) and were placed into Petri dishes until the parasitoids emerged. The parasitoids were placed in 96% ethanol for morphological and molecular determination.

### ﻿Morphological determination

According to Zuparko et al. (2011), the psyllid*Triozaeugeniae* is parasitized by *Tamarixiadahlsteni*; therefore, we employed Zuparko’s morphological description to identify the newly emerged parasitoids. Voucher specimens of the recovered parasitoids were deposited in the Beneficial Insects Collection of the Universidad Autónoma de Nuevo León (CIBE–UANL). All individuals followed the same diagnosis and, given the low number of specimens recovered, only one female was photographed with a scanning electronic microscope (JEOL JSM–6510LV) in order to illustrate its diagnostic characteristics.

### ﻿Barcoding determination

Genomic DNA was non-destructively isolated according to the protocol described by Giantsis et al. (2016). We extracted three individual specimens that corresponded to *T.dahlsteni*, two specimens of *T.triozae* and two of *T.schina*. Polymerase chain reaction (PCR) was carried out to amplify the DNA barcode region of the cytochrome oxidase subunit I (COI) using the LCO1490 (5’-GGTCAACAAATCATAAAGATATTGG-3’) and HCO2198 (5‘-TAAACTTCAGGGTGACCAAAAAATCA-3’) primers (Folmer et al. 1994). PCRs were performed in a 20 µl reaction volume: 2 µl of DNA, 2 µl of 10× Qiagen PCR buffer containing 15 mM MgCl2, 0.9 µl of each primer (10 um), 0.6 µl of dNTPs (25 mM each), 0.2 µl of (5 U/µl) Taq DNA Polymerase (Qiagen, Hilden, Germany), and 13.4 µl of H_2_O. PCR conditions were as follows: 94 °C for 3 min, followed by 40 cycles of 94 °C for 30 s, 52 °C for 1 min, 72 °C for 1 min with a final extension at 72 °C for 10 min. All PCR products were electrophoresed through an agarose gel (1%) and sequenced in both directions in an Applied Biosystems model 3500 automated sequencer in Lanbama–Ipicyt (San Luis Potosí, Mexico).

### ﻿Phylogenetic analysis

We employed the resulting COI sequences to reconstruct the phylogenetic relations of the emerged parasitoids. For this objective, we included COI sequences of all available species of the genus *Tamarixia*, which were downloaded from the BOLD Systems database and GenBank. All sequences were aligned in Mesquite ver. 3.70 (Maddison and Maddison 2021) with the program MUSCLE ver. 3 (Edgar 2004). Later, the alignment was used for the phylogenetic analysis inferred with the Maximum Likelihood method in the online server IQ-tree ver. 1.6.12 (Trifinopoulos et al. 2016), and the model GTR+F+I+G4 which was inferred with the function Model Finder (Kalyaanamoorthy et al. 2017). Branch support was obtained with the ultrafast bootstrap approximation (Hoang et al. 2018) with 10 000 replicates. Sequences generated in this study were deposited in GenBank.

## ﻿Results

### ﻿Morphological and barcoding determination

**Figures 1–3.**
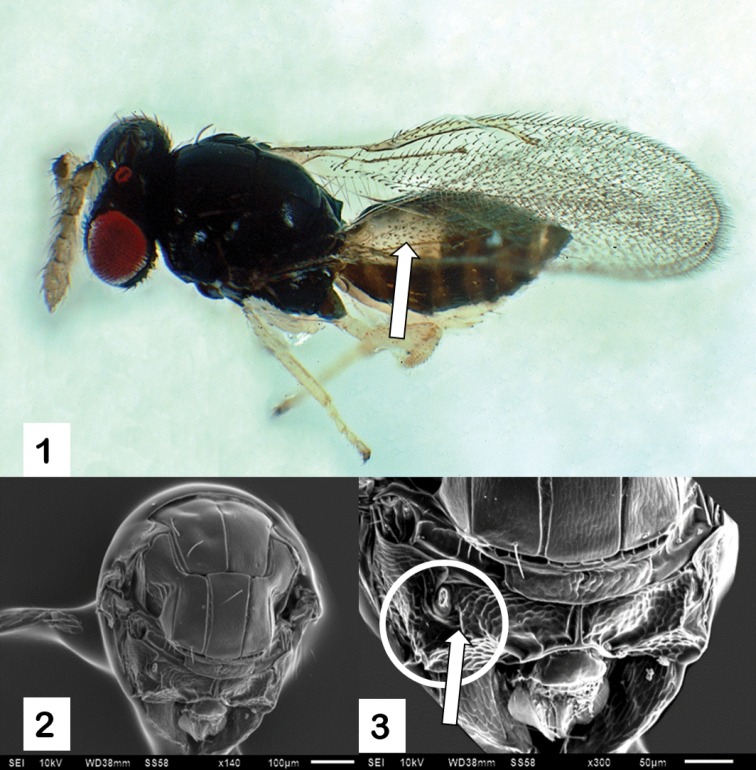
*Tamarixiadahlsteni***1** gaster (dorsal view), arrow pointing the yellow color extending to the apex of the second tergite **2** mesosoma (dorsal view) **3** arrow pointing paraspicular carina. Scale bars: 100 μm (**2**); 50 μm (**3**).

From the collected material for this study, seven females and seven males of the genus *Tamarixia* emerged. They agreed with the diagnosis of *T.dahlsteni*: the entire ventral surface of the gaster was yellow, and in the dorsal part the yellow color extending to, or slightly beyond, the apex of the second tergite (Fig. 1). The specimens had a paraspicular carina posteriorly bifurcated and located medial to the propodeal spiracle (Figs 2, 3). Regarding the barcoding determination, the sequences generated in this study represent the first barcoding evidence for the species *T.dahlsteni* (GenBank accession ON491415, ON491416, ON491417) and *T.schina* (GenBank accession ON548243, ON684328).

### ﻿Phylogenetic analysis

**Figure 4.**
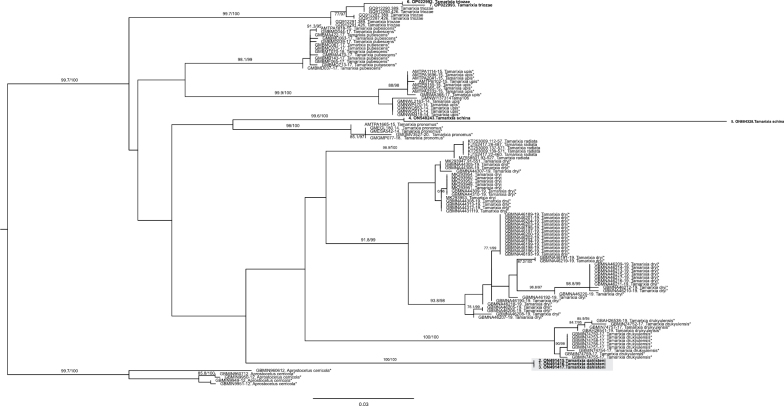
Phylogenetic tree showing the genetic relations between species of the genus *Tamarixia*. *Aprostocetuscerricola* was included as an outgroup and *Tamarixiadahlsteni* is highlighted in gray color. Bootstrap branch support ≥ 95% is shown above branches; samples in bold represent sequences generated in this study, while samples with an asterisk (*) were downloaded from the BOLD Systems database. The rest of the samples were downloaded from GenBank. BOLD Systems specimen records and GenBank accession numbers are shown before the species name.

Our sequence alignment contained 893 bp, and included sequences of the species *Tamarixiadrukyulensis* Yefremova and Yegorenkova, *Tamarixiadryi* Waterston, *Tamarixiapronomus* Walker, *Tamarixiapubescens* Nees, *T.radiata*, *T.triozae* and *Tamarixiaupis* Walker, obtained from public databases and those of *T.dahlsteni* and *T.schina* generated in this study. In the phylogenetic reconstruction, sequences of each species were clustered in individual subclades with high support (ultrabootstrap values >95) (Fig. 4). Therefore, the barcoding region appears to be useful for the molecular identification of the *Tamarixia* species included in this study. Our analysis did not resolve interspecific relations due to the low bootstrap support for interior branches. On the other hand, the presence of highly supported (ultrabootstrap values >95) intraspecific subclades within *T.drukyulensis* and *T.dryi* suggests intraspecific genetic structure.

## ﻿Discussion

According to the literature, seven of the 54 existing species of *Tamarixia* are reported from the Nearctic and Neotropical regions (Noyes 2022). Mexico spans both regions and, following this study, the number of *Tamarixia* species was updated to six. In addition, our phylogenetic analysis suggested the COI barcoding region to be a useful molecular marker for the distinction of *Tamarixia* species. The phylogeny obtained suggested intraspecific genetic structure for some of the species, which unveils the necessity of robust and wider phylogenetic analysis at the genus level.

The host for *T.dahlsteni* is the eugenia psyllid*Triozaeugeniae*. Both species were found in Australia associated with the ornamental tree *Syzygiumpaniculatum*, and in 1988 the psyllid was found for the first time in California, USA. The damage caused by the psyllid on *S.paniculatum* trees prompted a search for its natural enemies in Australia. As a result, the wasp *T.dahlsteni* was identified as the primary parasitoid of *T.eugeniae* and was later imported to the USA to control eugenia psyllid populations (Dahlsten et al. 1993). A similar case is the one of *Tamarixiaschina*. The wasp was reported as a natural enemy of the exotic psyllid*Calophyarubra* (Blanchard) which feeds on *Schinusmolle* trees (Álvarez-Zagoya and Cibrián-Tovar 1999). According to Zuparko et al. (2011), new undescribed *Tamarixia* species were found in both California and Florida, USA, parasitizing psyllid species which have potential distribution in Mexico.

In biological research, names of species are essential to ensure comparable results when working with model organisms (Pante et al. 2015), and in agriculture they are also required for biosecurity and quarantine concerns (Lyal et al. 2008). Notwithstanding, in some cases species identification is not an easy task and deep taxonomic studies are needed. For instance, in 2019 the misidentification of the eugenia psyllid*T.eugeniae* was uncovered by Taylor and Martoni (2020), who indicated that the true name of the species should be *Triozaadventicia* Tuthill. Taylor and Martoni mentioned that the two exotic species resemble each other, and only a detailed study based on a series of morphological characters and DNA barcoding supported the separation and validity of both species.

As already mentioned, psyllids are main hosts for the *Tamarixia* species and because of their possible broad dietary tolerance some species might migrate and disperse to new geographic regions (Percy et al. 2012). This might also promote the introduction and dispersion of exotic or new species of parasitoids in countries like Mexico. For example, *T.schina* apparently migrated to Mexico from California (Yefremova et al. 2014). According to the study by Percy et al. (2012), in the same region (California, USA), different species of psyllids attacked by some unidentified *Tamarixia* species exist, hosted by plants with potential distribution in Mexico. Moreover, in this country more than 114 species of psyllids (Méndez-Tobar 2015) exist, which may also represent possible hosts for exotic *Tamarixia* species. Therefore, the diversity of this genus might be currently underestimated in Mexico.

In agriculture, species identification protocols based on DNA represent powerful tools for the success of early detection programs, or monitoring of species (Lyal et al. 2008; Boykin et al. 2012; Poland and Rassati 2019). However, for some groups of insects, the lack of reference barcodes, errors in databases, scarcity of voucher specimens and presence of cryptic species represent strong limitations. As an example, recently a new *Tamarixia* species (*T.aguacatensis*) was described based on morphological characters, but because their sampling seems to be seasonally restricted (Yefremova et al. 2014), the generation of barcodes for further studies on their biology and phylogeny represents a challenge.

## ﻿Conclusion

Besides reporting the presence of *Tamarixiadahlsteni* in Mexico, we also provided barcodes that may be employed as a reference for further monitoring programs or studies about this economically important group of wasps. Moreover, our phylogenetic analysis suggests the need for a deeper and wider taxonomic revision of the genus.
